# Associated Factors of Hemorrhagic Transformation in Patients Undergoing Anterior Circulation Thrombectomy: Data from a Comprehensive Stroke Center in Vietnam

**DOI:** 10.3390/jcm15197426

**Published:** 2026-09-24

**Authors:** Dao Viet Phuong, Pham Quang Tho, Hoa Thi Truong, Nguyen Tien Dung, Pham Thuy Linh, Vu Thi Thom, Tran Cong Minh, Thanh N. Nguyen, Mai Duy Ton

**Affiliations:** 1 Faculty of Stroke and Cerebrovascular Diseases, VNU-University of Medicine and Pharmacy, Hanoi 10000, Vietnamthomtbk5@gmail.com (V.T.T.); 2 Stroke Center, Bach Mai Hospital, Hanoi 10000, Vietnam; 3 Department of Emergency and Critical Care Medicine, Hanoi Medical University, Hanoi 10000, Vietnam; 4 Nuffield Department of Clinical Neurosciences, University of Oxford, Oxford OX3 9DU, UK; 5 Boston Medical Center, 1 Boston Medical Center Place, Boston, MA 02118, USA

**Keywords:** hemorrhagic transformation, symptomatic intracranial hemorrhage, mechanical thrombectomy, acute ischemic stroke, clinical outcome

## Abstract

**Background/Objectives:** Hemorrhagic transformation (HT) is a common complication after mechanical thrombectomy. This study aimed to determine the incidence of and factors associated with HT, evaluate factors associated with sICH, and assess prognostic factors associated with 90-day functional outcome after anterior-circulation thrombectomy. **Methods:** We retrospectively analyzed 244 consecutive patients with acute ischemic stroke who underwent anterior circulation thrombectomy between June 2022 and May 2023. Clinical, laboratory, imaging, and treatment variables were compared according to HT, sICH, and 90-day functional outcome. Univariate and multivariable logistic regression analyses were performed. **Results:** HT and sICH occurred in 38.5% and 10.0% of patients, respectively. Cardioembolic etiology was independently associated with HT (adjusted odds ratio [aOR] 2.39, 95% confidence interval [CI] 1.31–4.36) and sICH (aOR 2.79, 95% CI 1.12–6.93). Successful reperfusion (TICI 2b–3) was independently associated with HT (aOR 2.51, 95% CI 1.09–5.77). Among patients with HT, younger age (aOR 0.86, 95% CI 0.78–0.95) and a lower 24-h National Institutes of Health Stroke Scale score (aOR 0.62, 95% CI 0.43–0.88) were independently associated with good functional outcome at 90 days. **Conclusions:** Cardioembolic etiology and successful recanalization were independently associated with HT, whereas cardioembolic etiology was independently associated with sICH. Among patients with HT, younger age and a lower 24-h neurological deficit were prognostic markers of better functional outcomes.

## 1. Introduction

Stroke is a leading cause of death and disability worldwide [1]. In Vietnam, the incidence is approximately 222 cases per 100,000 population, with 135,999 deaths recorded in 2019, underscoring stroke as a major disease burden and the need for improved diagnosis and treatment, particularly mechanical thrombectomy, to optimize patient outcomes [2,3].

Hemorrhagic transformation (HT) refers to the new appearance of intracranial hemorrhage in patients with acute ischemic stroke, as identified on follow-up brain imaging (CT or MRI) [4]. HT is a common complication among patients with acute ischemic stroke due to large-vessel occlusion undergoing mechanical thrombectomy [5]. Proposed mechanisms include disruption of the blood–brain barrier and a sudden increase in cerebral blood flow after reperfusion. HT increases the risk of clinical deterioration and may prolong hospital stay [6].

Prior studies evaluating patients with HT have identified cardiac thrombus, extensive parenchymal injury, and poor collateral status as factors that increase the risk of post-reperfusion hemorrhage [7,8]. However, data on HT in the Vietnamese population—which has a different distribution of stroke etiologies and a lower prevalence of cardioembolism—remain limited [9]. However, data on hemorrhagic complications following mechanical thrombectomy in the Vietnamese population remain limited. Therefore, the primary objective of this study was to determine the incidence of HT and identify factors associated with HT among patients with anterior-circulation large-vessel occlusion undergoing mechanical thrombectomy. The secondary objective was to evaluate factors associated with sICH. In addition, we evaluated prognostic factors associated with 90-day functional outcome, particularly among patients who developed HT.

## 2. Materials and Methods

### 2.1. Study Design and Participants

This observational, retrospective, longitudinal study included consecutive patients with acute ischemic stroke due to anterior circulation large-vessel occlusion (ICA or MCA) who underwent thrombectomy within 24 h of symptom onset at Bach Mai Hospital between June 2022 and May 2023. Indications for mechanical thrombectomy followed the 2019 American Heart Association/American Stroke Association (AHA/ASA) guidelines for the early management of patients with acute ischemic stroke [10]. Patients were excluded if they had acute ischemic stroke due to simultaneous anterior and posterior circulation occlusion, a pre-stroke modified Rankin Scale (mRS) score ≥ 2, pregnancy or breastfeeding, or a concomitant brain lesion (e.g., traumatic brain injury, intracranial or subarachnoid hemorrhage, or brain tumor). All patients were managed at the Stroke Center and underwent comprehensive clinical evaluation. Clinical variables included demographics (age, sex), vascular risk factors (hypertension, diabetes, atrial fibrillation), admission status (NIHSS score, vital signs), laboratory results, stroke etiology per the Trial of Org 10172 in Acute Stroke Treatment (TOAST) classification (large-artery atherosclerosis, cardioembolism, or undetermined etiology), and the modified Rankin Scale (mRS) score at 90 days. Treatment-related variables—including rate of intravenous thrombolysis, time from onset, onset-to-recanalization time, procedure duration, number of device passes, procedural outcomes, and adverse events—were extracted from the hospital’s interventional procedure records. NIHSS and mRS assessments were performed by trained, certified clinicians. Stroke onset time was recorded as the witnessed onset time when available; otherwise, the last-known-well time was used for unwitnessed or wake-up strokes.

Stroke etiology was classified according to the TOAST criteria based on the overall clinical, neuroimaging, vascular, and cardiac evaluation. All patients underwent 12-lead electrocardiography, transthoracic echocardiography, and carotid Doppler ultrasonography. In patients with a high clinical or imaging suspicion of a cardioembolic mechanism, additional investigations, including Holter electrocardiographic monitoring and/or transesophageal echocardiography, were performed when clinically indicated. Patients with evidence supporting a large-artery atherosclerotic mechanism and no identified cardiac source were classified as having large-artery atherosclerosis, whereas those with an identified cardiac source consistent with the index stroke were classified as having cardioembolic stroke. When more than one plausible mechanism was present and a single mechanism could not be confidently assigned, such as concomitant atrial fibrillation and relevant internal carotid or middle cerebral artery stenosis, the stroke was classified as being of undetermined etiology.

### 2.2. Imaging Procedures and Assessment

On admission, all patients underwent clinical evaluation followed by CT angiography (CTA) or MR angiography (MRA) before proceeding to mechanical thrombectomy. When indicated, intravenous thrombolysis was administered in the imaging suite prior to transfer to the angiography suite. The interventional procedure record captured time parameters, number of device passes, technique (e.g., balloon angioplasty, stent placement), complications, and angiographic outcome (modified Thrombolysis in Cerebral Infarction (mTICI) classification).

Follow-up neuroimaging was performed within 12–36 h after endovascular thrombectomy. Hemorrhagic transformation was identified on this protocol-defined follow-up examination by a trained, certified radiologist and classified according to the ECASS II criteria (four grades: HI1, HI2, PH1, PH2) and the Heidelberg classification [11,12,13]. Symptomatic intracranial hemorrhage (sICH) was defined as new hemorrhage on brain imaging accompanied by an increase in NIHSS score of ≥4 points from the pre-deterioration baseline, or an increase of ≥2 points on a single NIHSS item.

Additional CT or MRI was performed when clinically indicated, including in patients with neurological deterioration. For the primary analysis, HT ascertainment was restricted to hemorrhagic findings identified on the predefined 12–36-h follow-up examination; hemorrhagic findings first detected on subsequent imaging outside this window were not included.

### 2.3. Study Outcomes

The primary outcome was hemorrhagic transformation (HT) identified on follow-up neuroimaging within 12–36 h after endovascular thrombectomy. The secondary safety outcome was symptomatic intracranial hemorrhage (sICH). In addition, 90-day functional outcome was assessed using the modified Rankin Scale (mRS). A good functional outcome was defined as an mRS score of 0–3. Prognostic factors associated with good functional outcome were further evaluated among patients who developed HT.

### 2.4. Ethical Considerations

The Ethics Committee of Bach Mai Hospital approved the study under Decision Number 19/BM-HĐĐĐ, with the council’s activity code IRB-VN01019. Written consent was obtained from all patients and their families before participation. Our study has been conducted in adherence to the Declaration of Helsinki.

### 2.5. Sample Size Calculation

The sample size was originally calculated to estimate the proportion of hemorrhagic transformation (HT) among patients with acute ischemic stroke undergoing endovascular thrombectomy. Based on a previously reported HT rate of 18.8% following endovascular thrombectomy [14], an anticipated prevalence of approximately 18% was used. With a two-sided 95% confidence level (α = 0.05) and an absolute margin of error of 5%, the required sample size was calculated using the single-proportion formula, $n=Z_{1-\alpha /2}^{2}p\left(1-p\right)/d^{2}$, yielding a minimum required sample size of approximately 227 patients. The final study cohort included 244 patients. This prevalence-based calculation was intended to provide adequate precision for estimating the proportion of HT and was not designed to determine the sample size required for the multivariable regression analyses.

### 2.6. Statistical Analysis

Patients were stratified into two sets of comparison groups: HT versus no HT, and sICH versus no sICH. The overall prevalence of HT was assessed across the cohort. Clinical characteristics—including age, sex, admission blood pressure, NIHSS score, blood glucose, and vascular risk factors (hypertension, diabetes, obesity, smoking, prior stroke or transient ischemic attack, and pre-stroke mRS)—were compared between groups. Laboratory and imaging variables, including ASPECTS, occlusion location, thrombectomy procedural details, and HT grade (ECASS and Heidelberg criteria), were also analyzed. The 90-day mRS was evaluated for the overall cohort and for the HT subgroup.

Continuous variables were compared using Student’s t-test for normally distributed data and the Kruskal-Wallis test for non-normally distributed data; categorical variables were compared using the χ^2^ test.

Univariate and multivariate logistic regression analyses were used to identify independent predictors of HT and sICH. Variables with a *p*-value <0.20 in univariable analysis were considered as candidate variables for the multivariable logistic regression models. In addition, variables considered clinically relevant or previously established as important factors based on prior evidence were considered regardless of their statistical significance in univariable analysis. Clinical overlap and potential redundancy among candidate variables were also considered to avoid unnecessary model complexity. Given the limited number of sICH events (n = 24), the multivariable model for sICH was deliberately restricted to four clinically relevant covariates (admission NIHSS score, WBC count, ASPECTS, and cardioembolic etiology) to reduce the risk of overfitting.

Among patients with HT, clinical outcome was assessed at 90 days after stroke onset. Multivariate logistic regression was used to identify independent predictors of good functional outcome, defined as mRS 0–3. All analyses were performed using SPSS version 20.0 (IBM Corp., Armonk, NY, USA), with *p* < 0.05 considered statistically significant.

The extent of missing data was assessed for each major study variable and is reported as the number and percentage of missing observations. Complete-case analysis was performed for the primary analyses, and no imputation was conducted. Given the retrospective nature of the study, missing data were not assumed to be completely random. The potential impact of missing data on the study findings was considered in the interpretation of the results.

## 3. Results

Among the 244 patients, 94 (38.5%) developed HT and 24 (10.0%) developed sICH (Table 1). Patients with HT and those with sICH shared several features, including lower ASPECTS, a higher prevalence of cardioembolic etiology, and elevated white-cell and neutrophil counts. Focusing on the sICH group alone, which represents a more severe complication, admission clinical status was worse, as reflected by lower GCS and higher NIHSS scores, together with a significantly higher rate of ICA occlusion, compared with the non-sICH group. There was no significant difference in age, sex, medical history (including rate of anticoagulant use), or intervention timing between the HT and non-HT groups, or between the sICH and non-sICH groups.

Follow-up imaging within 12–36 h after thrombectomy was performed using MRI in 124 patients (50.8%) and CT in 120 patients (49.2%). The mean interval from the endovascular procedure to follow-up imaging was 1283.3 ± 372.1 min (approximately 21.4 ± 6.2 h). MRI was used in 50 patients with HT (53.2%) and 74 patients without HT (49.3%), with no significant difference in imaging modality between the two groups (*p* = 0.56).

Variables associated with HT and sICH on univariate analysis were entered into multivariate regression models (Table 2). For the sICH analysis, given the limited number of outcome events, a parsimonious multivariable logistic regression model was constructed to reduce model complexity and the risk of overfitting. Four covariates that were associated with sICH in univariable analysis (*p* < 0.05) and were considered clinically relevant were included: admission NIHSS score, WBC count, ASPECTS, and cardioembolic etiology. Admission GCS and ICA occlusion were not simultaneously included to limit model complexity and potential redundancy with measures of neurological severity and ischemic burden. Cardioembolic etiology was independently associated with both HT (aOR 2.39, 95% CI 1.31–4.36) and sICH (aOR 2.79, 95% CI 1.12–6.93; *p* = 0.03). Successful reperfusion (TICI 2b-3) was independently associated with HT (aOR 2.51, 95% CI 1.09–5.77) but not with sICH.

Although lower ASPECTS was observed among patients with HT and those with sICH in the univariable comparisons, ASPECTS did not remain independently associated with either HT (aOR 1.29, 95% CI 0.98–1.68; *p* = 0.07) or sICH (aOR 1.48, 95% CI 0.96–2.28; *p* = 0.08) after multivariable adjustment.

In an additional sensitivity analysis incorporating intravenous thrombolysis into the adjusted sICH model, intravenous thrombolysis was not significantly associated with sICH (aOR 0.41, 95% CI 0.12–1.36; *p* = 0.147), whereas cardioembolic etiology remained independently associated with sICH (aOR 2.89, 95% CI 1.15–7.25; *p* = 0.024). (Supplementary Table S1).

Successful reperfusion, defined as mTICI 2b–3, was achieved in 198 patients (81.1%), whereas 168 patients (68.9%) achieved mTICI 2c–3. Another sensitivity analysis using the more stringent mTICI 2c–3 threshold revealed that the association between reperfusion and HT was attenuated and was no longer statistically significant (aOR 1.33, 95% CI 0.71–2.51; *p* = 0.379), compared with the primary analysis using mTICI 2b–3 (aOR 2.51, 95% CI 1.09–5.77; *p* = 0.03). (Supplementary Table S2).

Among the 94 patients with HT, 57 (60.6%) achieved an mRS score of 0–3 and 34 (36.2%) achieved an mRS score of 0–2 at 90 days, and mortality following thrombectomy was 22.3% in this subgroup. We compared patients with a good functional outcome (mRS 0–3) and those with a poor outcome (mRS 4–6) (Table 3). The good-outcome group was younger and had better admission clinical status, reflected by higher GCS scores (14 vs. 13) and lower NIHSS scores both at admission (13 vs. 15) and at 24 h (10 vs. 18). The incidence of parenchymal hematoma (PH)-type HT was significantly higher in the poor-outcome group (70.3% vs. 22.8%).

These same variables were entered into a multivariate regression model to identify factors independently associated with good outcome among patients with HT after thrombectomy (Table 4). Age (aOR 0.86, 95% CI 0.78–0.95) and 24-h NIHSS score (aOR 0.62, 95% CI 0.43–0.88) were independent prognostic factors for good outcome in this subgroup. In a sensitivity analysis using mRS 0–2 as the definition of favorable functional outcome, younger age (aOR 0.87, 95% CI 0.78–0.97; *p* = 0.012) and lower 24-h NIHSS (aOR 0.55, 95% CI 0.38–0.81; *p* = 0.002) remained independently associated with favorable outcome (Supplementary Table S3).

## 4. Discussion

HT is a common complication after reperfusion therapy with mechanical devices. Prior studies have reported that more than one-third of patients with acute ischemic stroke due to large-artery occlusion undergoing endovascular thrombectomy develop HT. Our observed rate of 38.5% is consistent with this range [5].

HT after reperfusion therapy for acute ischemic stroke involves multiple mechanisms, including blood–brain barrier disruption, endothelial injury with increased vascular permeability, and reperfusion-induced cascades of oxidative stress, impaired protein synthesis, platelet activation, and basement-membrane degradation [6].

In this study, cardioembolic etiology was independently associated with both HT (aOR 2.39, 95% CI 1.31–4.36) and sICH (aOR 3.14, 95% CI 1.11–8.88), despite comparable rates of anticoagulant use between groups. Cardiac thrombi often occlude large arterial branches, producing extensive infarction. Poor collateral circulation in the setting of sudden thrombotic occlusion may also accelerate infarct progression, leading to rapid, irreversible injury to the brain parenchyma and blood–brain barrier. In addition, natural degradation of fibrin-rich, red-cell-predominant cardioembolic thrombi may permit distal migration of thrombus fragments, promoting infarct expansion and reperfusion injury [15,16]. Expert consensus has similarly identified cardioembolism as a significant risk factor for HT after acute ischemic stroke [17].

Studies evaluating the effect of cerebral revascularization on HT have yielded conflicting results. Kaesmacher et al., Wang et al., and Luo et al. reported a protective effect of successful revascularization, with lower rates of HT [18,19,20]. In contrast, Ciacciarelli et al. reported a trend toward a higher rate of HT after successful thrombectomy (TICI 2b-3) [21]. In our cohort, TICI 2b-3 recanalization was associated with a higher rate of HT; however, successful recanalization did not increase the rate of sICH (*p* > 0.05). This finding is consistent with Javed et al., who reported that TICI 2b-3 independently predicts HT but not sICH [22].

However, the association between successful reperfusion (mTICI 2b–3) and HT should be interpreted cautiously. In a sensitivity analysis using the more stringent mTICI 2c–3 threshold, the association was attenuated and no longer statistically significant (aOR 1.33, 95% CI 0.71–2.51; *p* = 0.379), indicating that this finding was sensitive to the definition of successful reperfusion. Although this attenuation raises the possibility that patients achieving mTICI 2b contributed to the association observed with the conventional mTICI 2b–3 threshold, our analysis does not establish that the association was specifically driven by this subgroup.

Several factors may nevertheless contribute to the observed association with the mTICI 2b–3 definition. Reperfusion of tissue with established ischemic and blood–brain barrier injury may predispose to hemorrhagic transformation, while baseline infarct burden, occlusion characteristics, collateral status, intravenous thrombolysis, number of thrombectomy passes, and treatment-selection factors may influence both reperfusion status and the risk of HT. Notably, patients who developed HT had lower baseline ASPECTS in the unadjusted analysis, whereas recanalization time was similar between groups, suggesting that baseline ischemic injury may have contributed to the observed relationship. Given the threshold-dependent finding, the retrospective observational design, and the possibility of residual confounding, the association between reperfusion status and HT should be considered hypothesis-generating and should not be interpreted as evidence that successful reperfusion itself causes HT.

Intravenous thrombolysis was less frequent among patients who developed sICH in the unadjusted analysis. However, this inverse association was attenuated and was no longer statistically significant after adjustment in a sensitivity analysis. Given the retrospective observational design and the limited number of sICH events, this finding should not be interpreted as evidence of a protective effect of intravenous thrombolysis and may reflect treatment-selection effects or residual confounding.

Several factors have been identified as predictors of clinical outcome after anterior circulation thrombectomy. Older age, higher NIHSS score at admission, and lower ASPECTS are associated with poor outcome at three months [23]. HT increases mortality and worsens clinical outcome in patients with acute ischemic stroke [24,25].

Data evaluating prognostic factors specifically in patients with HT remain limited. We analyzed a subgroup of 94 patients with HT to identify predictors of good functional outcome (mRS 0–3) at 90 days. Good outcome was more common among patients who were younger, had better admission clinical status (higher GCS and lower NIHSS scores), and had less severe hemorrhage (a higher proportion with Heidelberg class 1 and a lower proportion with PH-type hemorrhage). On multivariate analysis, two factors were independently associated with good outcome: age (aOR 0.86, 95% CI 0.78–0.95) and 24-h NIHSS score (aOR 0.62, 95% CI 0.43–0.88). Importantly, these associations remained significant in a sensitivity analysis using the more conventional mRS 0–2 threshold, suggesting that the principal prognostic findings were not dependent on the prespecified mRS 0–3 definition of favorable functional outcome.

Advancing age is associated with declining organ reserve, including reduced intracranial collateral capacity, diminished tissue reperfusion, weakened immune function, and greater susceptibility to post-interventional infection and other complications. The NIHSS score reflects the severity of neurological injury, with higher scores correlating with a greater risk of unfavorable prognosis after endovascular thrombectomy. Several studies have shown that, despite successful recanalization, advanced age and high NIHSS scores remain associated with poor outcome [26].

This study has several limitations. It was a retrospective, single-center study with a limited sample size, which may introduce selection bias and may not be generalizable to other populations; multicenter studies with larger cohorts and prospective designs are needed to validate these findings. In addition, the relatively small number of sICH events (n = 24) limited the complexity and precision of the multivariable analysis. To reduce the risk of overfitting, we restricted the sICH model to four clinically relevant covariates. Nevertheless, model instability cannot be completely excluded, and the adjusted estimates should therefore be interpreted cautiously. Otherwise, the use of both CT and MRI for follow-up imaging may have introduced imaging ascertainment bias because the two modalities may differ in their sensitivity for detecting small hemorrhagic lesions, although the distribution of MRI versus CT did not differ significantly between the HT and non-HT groups. Furthermore, HT ascertainment was restricted to the predefined 12–36-h post-procedural imaging window; therefore, delayed hemorrhagic transformations first appearing on subsequent imaging may not have been captured. Finally, several potentially relevant factors, including antiplatelet use, international normalized ratio, and collateral status, were not evaluated.

## 5. Conclusions

Hemorrhagic transformation is a common complication of endovascular thrombectomy in patients with anterior circulation large-vessel occlusion. Cardioembolic etiology and TICI 2b-3 recanalization were independently associated with HT, while cardioembolic etiology was independently associated with sICH. Among patients with HT, younger age and a lower 24-h NIHSS score were independently associated with good clinical outcome.

## Figures and Tables

**Table 1:** Characteristics of ischemic stroke patients with thrombectomy.

	HT (N = 94)	No HT (N = 150)	*p*	sICH (N = 24)	Non-sICH (N = 220)	*p*	Heidelberg 2–3 (N = 20)	Non-Heidelberg 2–3 (N = 224)	*p*
Age	63.7 ±12.7	64.2 ± 12.7	0.68	65.9 ±15.0	63.9 ± 12.3	0.46	64.1 ± 14.9	64.1 ± 12.4	0.98
Male sex	51 (54.3)	88 (58.3)	0.54	14 (58.3)	125 (56.6)	>0.99	13 (65.0)	126 (56.2)	0.45
Medical history									
Hypertension	45 (47.9)	82 (54.7)	0.30	9 (37.5)	118 (53.6)	0.13	8 (40.0)	119 (53.1)	0.26
Diabetes mellitus	15 (16.0)	25 (16.7)	0.88	6 (25.0)	34 (15.5)	0.25	8 (40.0)	32 (14.3)	0.01
Atrial fibrillation	39 (41.5)	49 (32.7)	0.16	12 (50.0)	76 (34.5)	0.13	8 (40.0)	80 (35.7)	0.70
Anticoagulants	19 (20.2)	22 (14.7)	0.26	3 (12.5)	38 (17.3)	0.78	4 (20.0)	37 (16.5)	0.75
Pre mRS 0–1	93 (98.9)	150 (100)	0.38	23 (95.8)	220 (100)	0.10	20 (100)	223 (99.6)	>0.99
Wake-up stroke	10 (10.6)	12 (8.0)	0.48	3 (12.5)	19 (8.6)	0.46	1 (5.0)	21 (9.4)	>0.99
GCS at admission	14 (13–15)	14 (13–15)	0.20	13 (12–14)	14 (13–15)	<0.01	13 (12–14)	14 (13–15)	0.02
NIHSS at admission	14 (12–16)	14 (10–16)	0.49	15 (14–18)	14 (10–16)	0.02	14.5 (14–16)	14 (10–16)	0.14
Systolic blood pressure at admission	143.3 ± 18.0	141.4 ±18.8	0.43	143.6 ± 21.0	142.1 ± 18.2	0.71	141.7 ± 21.2	142.3 ± 18.2	0.82
Blood glucose (mmol/L)	7.6 ± 3.2	7.6 ± 4.1	0.93	8.8 ± 4.4	7.4 ± 3.7	0.13	9.1 ± 5.0	7.4 ± 3.7	0.20
WBC	11.7 ± 3.9	10.5 ± 4.2	0.04	12.9 ± 4.9	10.8 ± 4.0	0.02	12.0 ± 4.5	10.9 ± 4.1	0.22
Neutrophil	75.4 ± 12.4	71.0 ± 12.8	0.01	80.9 ± 10.9	71.8 ± 12.7	<0.01	77.0 ± 13.1	72.3 ± 12.7	0.10
Brain imaging									
Occlusion vessels									
ICA	23 (24.5)	33 (22.0)	0.66	10 (41.7)	46 (20.9)	0.02	6 (30.0)	50 (22.3)	0.42
M1	45 (47.9)	80 (53.3)	0.41	8 (33.3)	117 (53.2)	0.07	8 (40.0)	117 (52.2)	0.29
Tandem	23 (24.5)	26 (17.3)	0.18	5 (20.8)	44 (20.4)	>0.99	6 (30.0)	43 (19.2)	0.25
M2	3 (3.2)	11 (7.3)	0.18	1 (4.2)	13 (5.9)	>0.99	0 (0.0)	14 (6.2)	0.61
ASPECTS	7 (6–8)	7 (7–8)	0.03	6.5 (6–7.5)	7 (6–8)	0.02	6.5 (6–8)	7 (6–8)	0.04
Thrombolysis	36 (38.3)	55 (36.4)	0.79	4 (16.7)	87 (39.4)	0.03	5 (25.0)	85 (37.9)	0.25
Thrombectomy procedure									
Onset to recanalization (minutes)	385.2 ± 183.2	386.7 ± 170.8	>0.99	411.5 ± 206.1	383.3 ± 172.0	0.49	370.0 ± 188.7	387.6 ± 174.5	0.60
Procedure duration (minutes)	43.1 ± 23.2	41.4 ± 26.9	0.22	47.5 ± 25.1	41.1 ± 25.5	0.27	45.9 ± 24.4	41.7 ± 25.6	0.33
No. of passes	2.3 ± 1.6	2.0 ± 1.4	0.16	2.8 ± 2.1	2.0 ± 1.4	0.06	2.2 ± 1.2	2.1 ± 1.5	0.43
No. of passes >3	15 (18.8)	19 (15.2)	0.57	5 (22.7)	29 (15.8)	0.38	4 (20.0)	34 (15.2)	0.53
First pass effect	30 (31.9)	42 (28.0)	0.51	7 (29.2)	65 (29.5)	0.97	6 (30.0)	66 (29.5)	0.96
Stenting	5 (5.8)	6 (4.6)	0.76	1 (4.3)	10 (5.2)	>00.99	1 (5.0)	11 (4.9)	>0.99
TICI 2b-3	77 (90.6)	93 (72.1)	<0.01	21 (91.3)	149 (78.0)	0.18	20 (100)	178 (79.5)	0.02
TICI 2c 3	69 (73.4)	99 (66.0)	0.22	18 (75.0)	150 (68.2)	0.49	18 (90.0)	150 (67.0)	0.03
MRI as follow-up imaging model	50 (53.2)	74 (49.3)	0.56	9 (37.5)	115 (52.3)	0.17	7 (35.0)	117 (52.2)	0.14
TOAST									
Large vessel atherosclerosis	41 (43.6)	88 (59.1)	0.03	9 (37.5)	120 (54.8)	0.11	8 (40.0)	121 (54.0)	0.23
Cardioembolism	50 (53.2)	52 (34.7)	0.01	15 (62.5)	88 (39.5)	0.03	12 (60.0)	90 (40.2)	0.09

mRS, Modified Rankin Scale; GCS, Glasgow Coma Scale; NIHSS, National Institutes of Health Stroke Scale; ICA, Internal Carotid Artery; M1, Middle Cerebral Artery segment 1; M2, Middle Cerebral Artery segment 2; ASPECTS, Alberta Stroke Program Early CT Score; WBC, White Blood Cell; TICI, Thrombolysis in Cerebral Infarction Scale; TOAST, Trial of ORG 10172 in Acute Stroke Treatment.

**Table 2:** Multivariate regression model for HT association.

	HT Prediction			sICH		
	aOR	95% CI	*p*	aOR	95% CI	*p*
Age	1.00	0.98–1.03	0.94			
Male	0.94	0.50–1.74	0.84			
GCS at admission	1.19	0.88–1.60	0.26			
NIHSS at admission	1.05	0.94–1.17	0.38	0.921	0.83–1.03	0.13
ASPECTS	1.29	0.98–1.68	0.07	1.476	0.96–2.28	0.08
TICI 2b-3	2.51	1.09–5.77	0.03			
WBC	0.96	0.88–1.04	0.27	0.910	0.83–1.00	0.05
Neutrophil	0.98	0.95–1.00	0.10			
Cardioembolism	2.39	1.31–4.36	0.01	2.790	1.12–6.93	0.03
Intravenous Thrombolysis	1.31	0.69–2.51	0.41			
ICA occlusion	1.15	0.57–2.31	0.70			

GCS, Glasgow Coma Scale; NIHSS, National Institutes of Health Stroke Scale; ASPECTS, Alberta Stroke Program Early CT Score; TICI, Thrombolysis in Cerebral Infarction Scale; WBC, White Blood Cell.

**Table 3:** Characteristics of HT patients.

	mRS 0–3 (N = 57)	mRS 4–6 (N = 37)	*p*
Age	60.6 ± 11.3	68.9 ± 13.2	<0.01
Male sex	33 (57.9)	18 (48.6)	0.38
Medical history			
Hypertension	29 (50.9)	16 (43.2)	0.47
Diabetes mellitus	6 (10.5)	9 (24.3)	0.07
Atrial fibrillation	23 (40.4)	17 (45.9)	0.59
Anticoagulants	13 (22.8)	7 (18.9)	0.65
Pre-mRS 0–1	56 (98.2)	37 (100)	>0.99
Wake-up stroke	6 (10.5)	3 (8.1)	>0.99
GCS at admission	14 (13–15)	13 (12–14)	<0.01
NIHSS at admission	13 (10–15)	15 (14–18)	<0.01
NIHSS 24 h	10 (6–12)	18 (14–23.5)	<0.01
Systolic blood pressure at admission	143.4 ± 15.7	143.0 ± 21.9	0.91
Blood glucose	7.1 ± 2.3	8.4 ± 4.1	0.08
Brain imaging			
Occlusion vessels			
ICA	13 (22.8)	10 (27.0)	0.64
M1	26 (45.6)	19 (51.4)	0.59
Tandem	16 (28.1)	7 (18.9)	0.31
M2	2 (3.5)	1 (2.7)	>0.99
ASPECTS	7 (6–8)	7 (6–8)	0.18
WBC	11.2 ± 3.3	12.2 ± 4.6	0.46
Neutrophil	74.6 ± 11.7	75.8 ± 14.5	0.42
Thrombolysis	26 (45.6)	10 (27.0)	0.07
Thrombectomy procedure			
Onset to recanalization	379.1 ± 187.5	385.4 ± 180.5	0.67
Procedure duration	41.8 ± 22.5	43.9 ± 23.3	0.72
No. of passes	2.1 ± 1.4	2.54 ± 1.9	0.23
No. of passes > 3	11 (19.3)	8 (21.6)	0.78
First pass effect	19 (33.3)	11 (29.7)	0.71
Stenting	3 (5.3)	2 (5.4)	>0.99
TICI 2b-3	51 (89.5)	34 (91.9)	>0.99
TOAST			
Cardioembolism	30 (52.6)	21 (56.8)	0.70
Heidelberg 1 classification	53 (93.0)	21 (56.8)	<0.01
PH classification	13 (22.8)	26 (70.3)	<0.01

mRS, Modified Rankin Scale; GCS, Glasgow Coma Scale; NIHSS, National Institutes of Health Stroke Scale; ICA, Internal Carotid Artery; M1, Middle Cerebral Artery Segment 1; M2, Middle Cerebral Artery Segment 2; ASPECTS, Alberta Stroke Program Early CT Score; WBC, White Blood Cell; TOAST, Trial of ORG 10172 in Acute Stroke Treatment; PH, Parenchymal Hemorrhage.

**Table 4:** Multivariate regression analysis for good outcomes (mRS 0–3) among HT patients.

Factors	aOR	95% CI	*p*
Age	0.86	0.78–0.95	<0.01
Male	0.16	0.02–1.20	0.07
GCS at admission	0.68	0.33–1.40	0.29
NIHSS at admission	0.68	0.44–1.06	0.09
NIHSS at 24 h	0.62	0.43–0.88	0.01
ASPECTS	1.95	0.86–4.44	0.11
TICI 2b–3	0.39	0.02–10.24	0.57
TOAST Cardioembolism	0.54	0.09–3.30	0.51
Heidelberg 1 classification	0.09	0.01–1.40	0.09
PH classification	3.47	0.61–19.75	0.16

GCS, Glasgow Coma Scale; NIHSS, National Institutes of Health Stroke Scale; ASPECTS, Alberta Stroke Program Early CT Score; TICI, Thrombolysis in Cerebral Infarction Scale; TOAST, Trial of ORG 10172 in Acute Stroke Treatment; PH, Parenchymal Hemorrhage.

## Data Availability

The datasets generated during and/or analyzed during the current study are available from the corresponding author on reasonable request.

## Supplementary Materials

The following supporting information can be downloaded at: https://www.mdpi.com/article/10.3390/jcm15197426/s1.
