# Supplementary material for: Associated Factors of Hemorrhagic Transformation in Patients Undergoing Anterior Circulation Thrombectomy: Data from a Comprehensive Stroke Center in Vietnam
Source: J Clin Med. 2026 Sep 24;15(19):7426. doi: 10.3390/jcm15197426 (PMC13666232; doi:10.3390/jcm15197426)
Supplement: Supplementary file 1 [file jcm-15-07426-s001.zip › jcm-4543219-supplementary.pdf]

Supplementary Table S1. Sensitivity analysis of factors associated with symptomatic intracranial hemorrhage after inclusion of intravenous thrombolysis in the multivariable model

	**sICH**		
	aOR	95% CI	p
NIHSS at admission	.94	.85-1.05	.27
ASPECTS	1.35	.82-.99	.04
WBC	.90	.87-2.10	.19
Cardioembolism	2.89	**1.15-7.25**	**.02**
Intravenous Thrombolysis	.41	**.12-1.36**	**.15**

Supplementary Table S2. Sensitivity analysis of factors associated with hemorrhagic transformation using mTICI 2c–3 as the threshold for successful reperfusion.

	**HT prediction**		
	aOR	95% CI	p
Age	1.00	.98 – 1.02	.98
Male	.94	.54 – 1.86	>.99
GCS at admission	1.18	.88 – 1.58	.26
NIHSS at admission	1.04	.94 - 1.16	.43
ASPECTS	1.21	.93 – 1.57	.16
TICI 2c-3	**1.33**	**.71 – 2.51**	**.38**
WBC	.97	.90 – 1.05	.45
Neutrophil	.98	.95 – 1.00	.10
Cardioembolism	**2.72**	**1.50 – 4.91**	**.001**
Intravenous Thrombolysis	1.37	.73 – 2.60	.33
ICA occlusion	1.12	.56 – 2.24	.75

Supplementary Table S3. Sensitivity analysis of factors associated with 90-day favorable functional outcome using mRS 0–2 among patients with hemorrhagic transformation.

**Factors**	**aOR**	**95% CI**	**p**
Age	.87	**.78-.97**	**.012**
Male	1.66	.18-15.72	.66
GCS at admission	.92	.38-2.27	.86
NIHSS at admission	1.05	.64-1.73	.85
NIHSS at 24 hours	.55	**.38-.81**	**.002**
ASPECTS	1.42	.59-3.43	.43
TICI 2b-3	1.25	.08-21.77	.88
TOAST Cardioembolism	.93	.11-7.63	.94
Heidelberg 1 classification	1.83	.08-42.37	.71
PH classification	7.47	.67-83.59	.10
